# Enhancement of Biomimetic Enzymatic Mineralization of Gellan Gum Polysaccharide Hydrogels by Plant-Derived Gallotannins

**DOI:** 10.3390/ijms21072315

**Published:** 2020-03-27

**Authors:** Timothy E. L. Douglas, Julia K. Keppler, Marta Vandrovcová, Martin Plencner, Jana Beranová, Michelle Feuereisen, Bogdan V. Parakhonskiy, Yulia Svenskaya, Vsevolod Atkin, Anna Ivanova, Patrick Ricquier, Lieve Balcaen, Frank Vanhaecke, Andreas Schieber, Lucie Bačáková, Andre G. Skirtach

**Affiliations:** 1 Nano-Biotechnology Group, Department of Biotechology, Faculty of Bioscience Engineering, Ghent University, 9000 Ghent, Belgium; Bogdan.parakhonskiy@UGent.be (B.V.P.); Andre.Skirtach@UGent.be (A.G.S.); 2 Engineering Department., Lancaster University, Gillow Avenue, Lancaster LA1 4YW, UK; 3 Materials Science Institute (MSI), Lancaster University, Lancaster LA1 4YW, UK; 4 Division of Food Technology, Kiel University, 24118 Kiel, Germany; julia.keppler@wur.nl; 5 Laboratory of Food Process Engineering, Wageningen University & Research AFSG, 6708 PB Wageningen, The Netherlands; 6 Department of Biomaterials and Tissue Engineering, Institute of Physiology of the Czech Academy of Sciences, 142 00 Prague, Czech Republic; Marta.Vandrovcova@fgu.cas.cz (M.V.); martin.plencner@gmail.com (M.P.); Lucie.Bacakova@fgu.cas.cz (L.B.); 7 Department of Genetics and Microbiology, Charles University in Prague, 116 36 Prague, Czech Republic; BeranovaJ@seznam.cz; 8 Department of Nutritional and Food Sciences, University of Bonn, D-53012 Bonn, Germany; mfeuerei@uni-bonn.de (M.F.); schieber@uni-bonn.de (A.S.); 9 FSRC “Crystallography and photonics” RAS, 119333 Moscow, Russia; ani@ns.crys.ras.ru; 10 Institute of Nanostructures and Biosystems, Saratov NG Chernyshevskii State Univ, 83 Astrakhanskaya St, 410012 Saratov, Russia; yulia_svenskaya@mail.ru (Y.S.); ceba91@list.ru (V.A.); 11 Omnichem NV, 9230 Wetterem, Belgium; Patrick.Ricquier@EU.AjiBio-Pharma.com; 12 Department of Chemistry, Ghent University, Krijgslaan 281, 9000 Ghent, Belgium; Lieve.Balcaen@UGent.be (L.B.); Frank.Vanhaecke@UGent.be (F.V.); 13 Centre for Nano- and Biophotonics, Ghent University, 9000 Ghent, Belgium

**Keywords:** mineralization, polyphenol, composite, protein-polyphenol interaction, gellan gum, enzyme

## Abstract

Mineralization of hydrogel biomaterials with calcium phosphate (CaP) is considered advantageous for bone regeneration. Mineralization can be both induced by the enzyme alkaline phosphatase (ALP) and promoted by calcium-binding biomolecules, such as plant-derived polyphenols. In this study, ALP-loaded gellan gum (GG) hydrogels were enriched with gallotannins, a subclass of polyphenols. Five preparations were compared, namely three tannic acids of differing molecular weight (MW), pentagalloyl glucose (PGG), and a gallotannin-rich extract from mango kernel (*Mangifera indica* L.). Certain gallotannin preparations promoted mineralization to a greater degree than others. The various gallotannin preparations bound differently to ALP and influenced the size of aggregates of ALP, which may be related to ability to promote mineralization. Human osteoblast-like Saos-2 cells grew in eluate from mineralized hydrogels. Gallotannin incorporation impeded cell growth on hydrogels and did not impart antibacterial activity. In conclusion, gallotannin incorporation aided mineralization but reduced cytocompatibility.

## 1. Introduction

To adapt hydrogels for applications in bone regeneration, they are enriched with a mineral phase, most commonly a form of calcium phosphate (CaP) (for a review, see [1]). One biomimetic mineralization method is the incorporation of alkaline phosphatase (ALP), the enzyme responsible for mineralization of bone tissue, followed by incubation in a mineralization solution of calcium glycerophosphate (CaGP). For example, ALP-mediated mineralization of gellan gum (GG) hydrogels with CaP reinforced the hydrogel mechanically and promoted the adhesion and growth of bone-forming cells, which is a pre-requisite for new bone formation [2].

Hence, modifications of a hydrogel to increase its mineralizability are desirable. An advantage of using hydrogels is the ease of incorporation of water-soluble biomolecules inside the hydrogel polymer network. Strategies to increase mineralizability include the incorporation of calcium-binding biomolecules [2,3] or phosphate-binding biomolecules [1,4]. The result is an increase in the intrahydrogel concentrations of calcium and phosphate ions, which in turn promotes CaP precipitation. It can be noted that ALP itself has been shown to increase the local inorganic phosphate concentration [5], but its combination with CaP results in the formation of hybrid organic–inorganic materials [6] reported to promote cell growth.

The mineralization of synthetic and natural hydrogels has been studied [7,8,9,10,11]. Synthetic hydrogels generally have better defined chemical structures, but often lack the functional groups with natural affinity for calcium (or phosphate) ions shown by natural polymers, such as alginate and GG. For the mineralization, various biomolecules have been used, including polyphenols, which have been successfully used for prevention and treatment of osteoporosis due to their protective effects on the bone mineral density [12,13]. In addition, polyphenols promoted biological mineralization of Ti6Al4V alloy by deposition of hydroxyapatite by mesenchymal stem cells cultured on this implant material, which is currently used in clinical practice [14]. In our earlier studies, polyphenols promoted mineralization of chitosan and gellan gum hydrogels (i.e., other materials that are promising for bone tissue engineering [15,16]. In this regard, polyphenols—plant-derived biomolecules present in plant cell walls [17]—are good candidates. It should be noted that certain polyphenols are known to display affinities for divalent metal ions, such as calcium [16]. One class of polyphenols known to bind calcium ions are the gallotannins [18]. Gallotannins consist of a glucose core esterified with gallic acid. The number of gallic acid units can range from one to more than ten [19]. Gallotannins are commonly extracted from plant seeds, such as mango kernels [20]. Typical gallotannins include decagalloyl glucose, more commonly known as tannic acid, and pentagalloyl glucose (PGG). Tannic acid is a well-known crosslinker in the leather industry through non-covalent interactions with collagen molecules. Tannic acid and PGG have been used to crosslink protein-based biomaterials [21,22].

The ability of gallotannins to promote hydrogel mineralization remains rather unexplored. Phlorotannins, another type of tannins derived from brown algae, have been reported to enhance osteogenic differentiation of mouse MC3T3E1 preosteoblasts, including a higher calcium concentration in these cells [23]. In our earlier study, Seanol(^®^), a seaweed extract rich in phlorotannins, induced mineralization of GG with CaP [16]. On the other hand, procyanidins (i.e., condensed tannins) prevented the calcification of elastin scaffolds for vascular tissue engineering, which was (besides other reasons) explained by direct blocking of the mineral nucleation sites in elastin fibers by procyanidins [24].

A further beneficial property of polyphenols is their antibacterial activity [25]. Due to the increasing prevalence of antibiotic-resistant bacteria, endowing biomaterials for implantation with antibacterial properties is desirable. Since gallotannins have shown antibacterial activity [14,26], they are expected to be promising antibacterial materials.

In this study, ALP-loaded GG hydrogels were enriched with gallotannins in order to enhance their mineralization. Five gallotanin preparations were used. We compared three tannic acids of differing molecular weight (MW) and PGG content with the brand names ALSOK2, ALSOK4 and Brewtan F (BTF) (see Section 3.1), along with PGG and a gallotannin-rich extract from mango kernel (*Mangifera indica* L.), which is known to contain a mixture of different gallotannins. As mentioned above, the incorporation of seaweed-derived polyphenols (phlorotannins) promoted hydrogel mineralization and endowed antibacterial activity in our previous work [16].

The ability of gallotannins to promote hydrogel mineralization was evaluated by calculating the dry mass percentage (i.e., the mass percentage of mineralized hydrogels attributable to newly formed minerals and polymers and not water). This served as a measure of mineral formation. In addition, amounts of elemental Ca and P in hydrogels as a result of mineralization were determined by inductively coupled plasma optical emission spectroscopy (ICP-OES). Further physicochemical characterization of formed CaP mineral included scanning electron microscopy (SEM), X-ray diffraction (XRD), and Fourier transform infrared spectroscopy (FTIR).

To evaluate electrostatic effects between protein and gallotannins and the addition of CaCl_2_, zeta-potential analyses were conducted in three interaction media (Table 1), namely water, CaCl_2_ solution, and GG/CaCl_2_ solution. To further explore the cross-linking ability of gallotannins with ALP, their interaction was followed by fluorescence analysis, and the aggregation potential of these molecules was observed by dynamic light scattering (DLS) in an interaction medium containing GG and CaCl_2_ (Table 2).

In addition, mineralized hydrogels were characterized by means of cell biology assays using human Saos-2 osteoblast-like cells. Growth of cells in eluates from mineralized hydrogels was analyzed using a real-time cell analyzer. Direct cell growth on mineralized hydrogels was evaluated using the standard 3-(4,5-dimethylthiazol-2-yl)-5-(3-carboxymethoxyphenyl)-2-(4-sulfophenyl)-2H-tetrazolium) (MTS) assay. Microbiological assays using *Escheria coli* (*E. coli*) were conducted by incubating mineralized hydrogels in bacterial suspensions followed by cultivation of the suspensions on agar to assess bacterial growth.

It was hypothesized that gallotannins would enhance hydrogel mineralization and endow antibacterial properties to mineralized hydrogels. It was also hypothesized that different gallotannins would interact differently with ALP.

## 2. Results and Discussion

### 2.1. Materials

**Figure 1:**
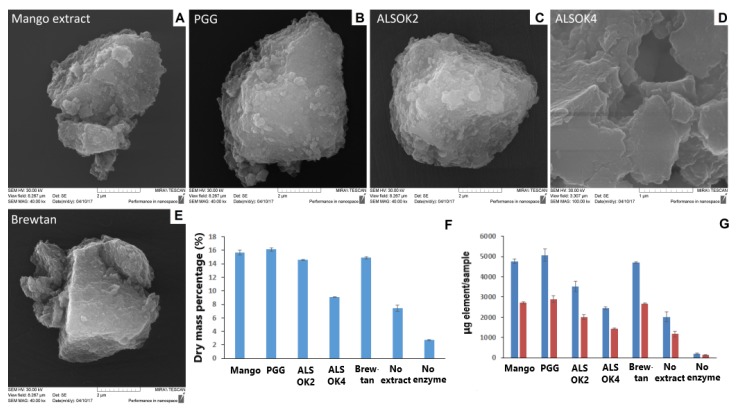
(**A**–**E**) Scanning electron microscopy (SEM) images of mineralized hydrogels containing different gallotannin preparations, (**F**) Dry mass percentage of mineralized hydrogels containing different gallotannin preparations (*n* = 3). (**G**) Inductively coupled plasma optical emission spectroscopy (ICP-OES) determination of amounts of elemental Ca (blue) and P (red) in mineralized hydrogels containing different gallotannin preparations (*n* = 3).

SEM images of mineralized hydrogels (Figure 1A–E) demonstrated the presence of inorganic deposits, which strongly suggested that mineral formation had taken place. Calculation of dry mass percentage (Figure 1F) demonstrated that values were clearly higher in the presence of the enzyme ALP. All gallotannin preparations increased dry mass percentage values, indicating that the presence of gallotannin promoted the mineral formation. Inductively Coupled Plasma-Optical Emission Spectroscopy (ICP-OES) measurements of elemental Ca and P in mineralized hydrogels (Figure 1G) largely confirmed the results of dry mass percentage measurements and the presence of mineral deposits suggested by SEM results. Differences were observed between sample groups. Mango extract, PGG, and Brewtan were the most successful at promoting mineralization, whereas ALSOK2, and in particular ALSOK4, promoted mineralization to a markedly lower extent.

**Figure 2:**
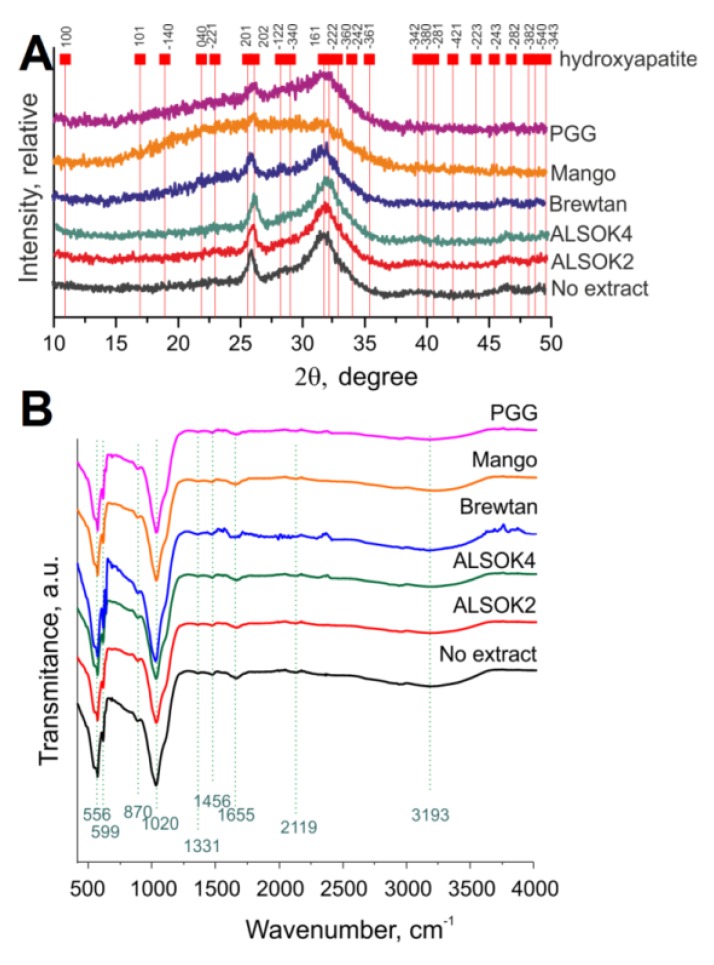
(**A**) X-ray diffraction (XRD) analysis of mineralized hydrogels containing different gallotannin preparations. Peaks indicated with red squares correspond to the hydroxyapatite phase. The Miller indices of each peak are highlighted on top. (**B**) FTIR analysis of mineralized hydrogels containing different gallotannin preparations.

XRD spectra (Figure 2A) demonstrated the presence of both calcium-deficient hydroxyapatite (CDHA) and amorphous CaP. Peaks characteristic for hydroxyapatite were observed at 2θ values of 26 and 32. Clearly, the mineral formed was not highly crystalline. In the case of samples containing mango extract, the CaP formed was markedly less crystalline. The reasons for this remain unclear. FTIR (Figure 2B) demonstrated the presence of CDHA in all samples.

### 2.2. Interactions between ALP and Gallotannins

**Figure 3:**
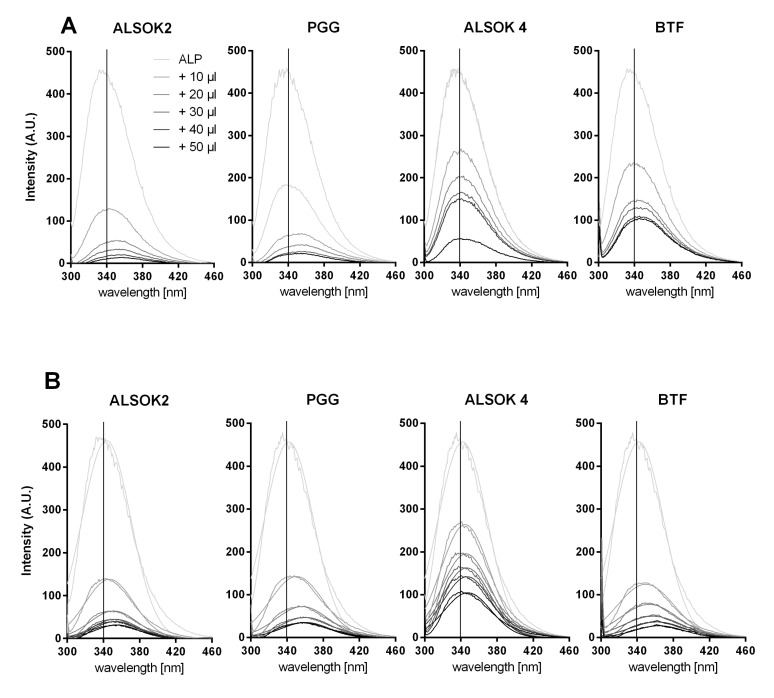

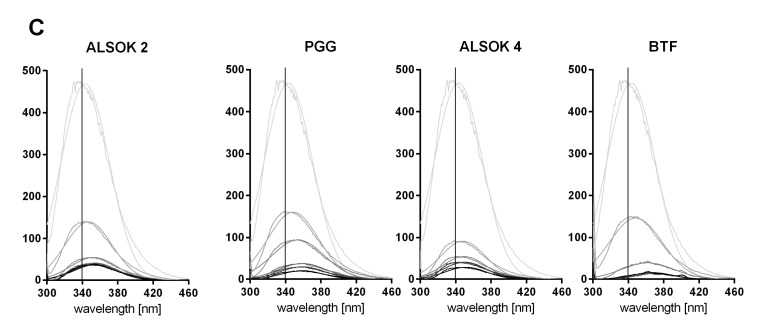
(**A**) Fluorescence emission spectra between 300 and 460 nm at 294 nm extinction of alkaline phosphatase (ALP) in water with increasing concentrations of gallotannins dissolved in dimethyl sulfoxide (DMSO). (**B**) Spectra between 300 and 460 nm with excitation at 294 nm of ALP + CaCl_2_, with increasing concentrations of tannins. (**C**) Spectra between 300 and 40 nm with excitation at 294 nm of ALP + CaCl_2_ + gellan gum (GG), with increasing concentrations of gallotannins. The vertical line at 340 nm was added to facilitate optical comparison of the different spectra.

**Figure 4:**
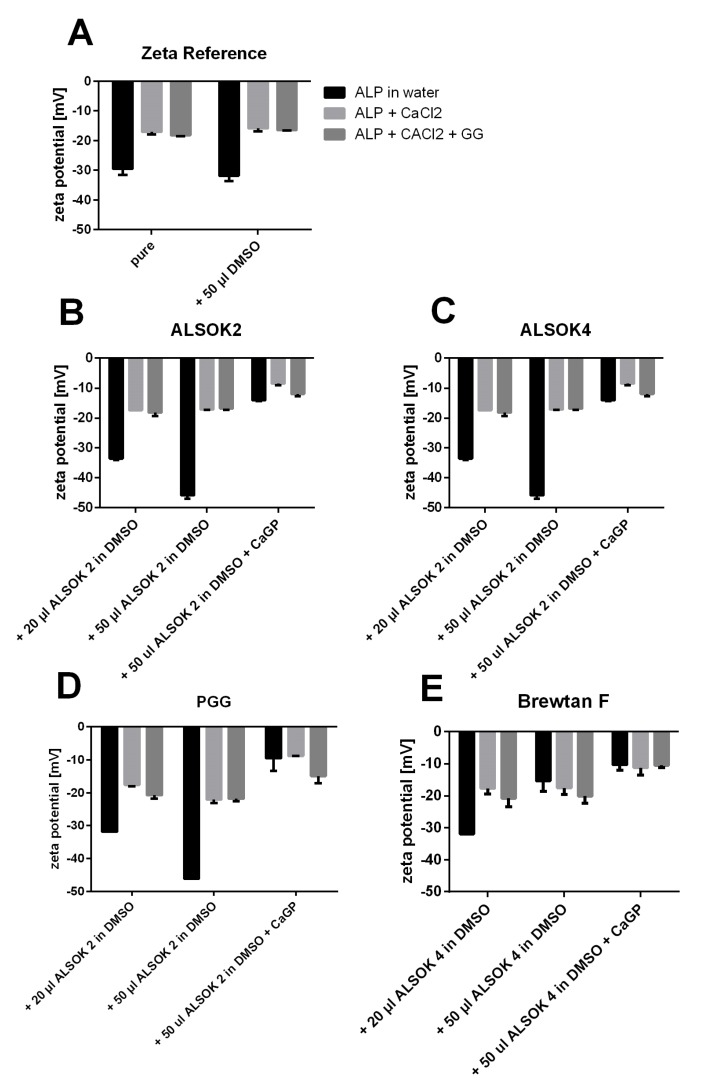
Zeta potential (mV) of ALP in water, ALP with CaCl_2_, and ALP with CaCl_2_ and GG with 20 or 50 µL of gallotannins in DMSO and with CaGP: (**A**) without gallotannins; (**B**) ALSOK2; (**C**) ALSOK4; (**D**) PGG; (**E**) Brewtan F. In all cases, *n* = 3.

The interactions between ALP and gallotannins were analyzed in the three different environments (Table 1) and in the concentrations used for the hydrogels. Non-covalent interactions between gallotannins and ALP were followed by fluorescence quenching analysis (Figure 3 and Figure 4), while protein cross-linking was also assessed by size changes via DLS and electrostatic complexes were evaluated using zeta potential measurements before and after ligand addition (Table 2).

The fluorescence intensity of the aromatic amino acid tryptophan (Trp) in ALP was followed at 294 nm excitation to avoid a strong overlap with tyrosine (excitation maximum at 280 nm). 

The Trp fluorescence of the ALP was significantly quenched after the addition of gallotannins, which hints at non-covalent interactions (Figure 3). The corresponding fluorescence emission maximum of ALP in water (interaction solution A) was continuously red-shifted from 340 nm, with rising concentration of gallotannins in the order ALSOK2 > PGG > Brewtan F > ALSOK4 (i.e., maximum shift by 19, 16, 11, and 9 nm, respectively) (Figure 3a). Similarly, for solutions of ALP with CaCl_2_ (interaction solution B) (Figure 3B) and ALP with CaCl_2_ and GG (interaction solution C) (Figure 3C), bathochromic shifts were observed, with ALSOK4 always showing the lowest wavelength shift. However, in interaction solution C, whose composition was most similar to that of the hydrogels, as it contained CaCl_2_ and GG, the red shift was in the order Brewtan F ≈ PGG > ALSOK2 > ALSOK4.

The photophysical properties of Trp are influenced by changes in the polarity of its environment, for example caused by non-covalent interactions [27,28]. A bathochromic shift indicates a more hydrophilic environment and complete denatured proteins show a maximum red shift due to increased solvent accessibility to Trp and the local electrostatic distribution changes [29,30]. Since the ALP-dimethyl sulfoxide (DMSO) spectra showed neither an emission maximum shift nor fluorescence quenching effect, any influence of DMSO on the protein conformation can be excluded. It is more likely that the shift was caused by hydrogen binding or other non-covalent interactions of the ALP with gallotannins.

**Figure A1:**
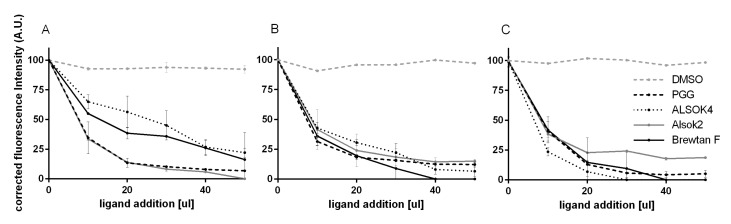
Relative fluorescence intensity of (**A**) ALP in water, (**B**) ALP + CaCl_2_ in water, and (**C**) ALP + CaCl_2_ + GG in water with increasing concentrations of gallotannins dissolved in DMSO after correction for inner-filtering effects. In all cases, *n* = 3.

The relative quenching of the Trp fluorescence at the 340 nm emission wavelength was further corrected for inner filter effects, which are caused by the increasing addition of the tannins absorbing the excitation and emission wavelength [28]. The gallotannins ALSOK 2 and PGG reacted most strongly with pure ALP in water (Figure A1a), whereas ALSOK 4 and Brewtan F resulted in a less pronounced fluorescence quenching effect. It is evident that the addition of CaCl_2_ (Figure A1B) increased the fluorescence quenching effect of these two gallotannins. Similarly, the addition of CaCl_2_ combined with GG (Figure A1) also affected the gallotannin–protein interaction positively, with the exception of ALSOK 2. There was no direct correlation between the bathochromic shift and the fluorescence quenching effect. It can be assumed that the red shift is also dependent on the number of hydroxyl groups and the gallotannin structure itself [27]. It was clear that DMSO had no effect on fluorescence quenching.

The non-covalent interaction of tannins or other polyphenols is a well-known process often followed by fluorescence quenching [31,32]. The binding described effect is primarily driven by hydrophobic attraction of the aromatic polyphenol rings to hydrophobic patches on the protein. In particular, PGG was found to interact by pi-stacking of aromatic groups between proteins and gallotannins [33]. These interactions can be further stabilized by hydrogen bonds to neighboring amino acids or to the protein backbone [34,35,36]. This often results in conformational changes of the protein, activity loss of enzymes, and protein aggregation, depending on the gallotannin/protein ratio. Previous results of the interaction of ALP with phlorotannins found a less strong reaction between the phlorotannins and the ALP [16], although such a comparison is difficult, since the experiments were conducted using similar ALP/polyphenol mass ratios. Differences in molecular weight between the phlorotannins in the aforementioned study and the gallotannins used in the present study were not taken into account. Furthermore, the phlorotannins used in the aforementioned study were more poorly defined (i.e., not all phlototannins could be identified and their relative proportions in the preparation were not determined) and heterogeneous than the gallotannin preparations used in the present study.

The addition of CaGP to all solutions resulted in gelling and precipitation, and therefore it was no longer possible to analyze fluorescence.

To assess electrostatic effects of the addition of CaCl_2_ and CaGP to ALP and tannin complexes, the zeta potential was analyzed (Figure 4). The zeta potential of ALP in water was approximately −30 mV. The addition of CaCl_2_ and GG decreased the zeta potential significantly to −20 mV (Figure 4A), probably due to electrostatic effects between positively charged Ca^2+^ and the negatively charged ALP. Generally, the addition of polyphenols to ALP in water resulted in an increased zeta potential of the complex, which is typically observed for non-covalent interactions between these two substances. This was, however, not observed for Brewtan F (Figure 4a). The addition of CaCl_2_ always resulted in a significant reduction of the zeta potential, with the exception of Brewtan F. This may be linked to the observation that the addition of CaCl_2_ increased the interaction between polyphenols and ALP (Figure 3). The reasons remain unclear. One can speculate that cross-linking effects occur, possibly because Ca^2+^ ions form ionic bridges between polyphenols and ALP. Zeta potential measurements indicated electrostatic interactions between Ca^2+^ and ALP (Figure 4). Polyphenols have been reported to show affinity for Ca^2+^ [18]. CaGP had no further significant effect on the charge.

Dynamic light scattering (DLS) was used to assess the aggregation effect of the non-covalent complexation. The z-average (intensity based harmonic mean of the particle size distribution) is a reliable measure for changes in particle size distributions, although distributions with a polydispersity index (PDI) > 0.7 are probably too polydisperse for proper analysis.

The z-average of ALP in interaction solution C without DMSO was approximately 82 nm, the addition of 20 µL DMSO had no effect on the z-average, although the addition of 50 µL DMSO increased the ALP diameter to 100 nm (Table 2). 

Addition of 50 µL of the different gallotannins to the ALP and CaCl_2_ solution led to different results. The addition of Brewtan F to ALP resulted in a significant increase of aggregate size up to 207 nm. PGG formed smaller aggregates of approximately 166 nm, whereas ALSOK2 and ALSOK 4 resulted in 148 nm and 91 nm aggregates. Generally, the addition of 50 µL gallotannin solution had a stronger effect on the aggregate size than the smaller volume of 20 µL. ALP in DMSO and ALSOK2 was extremely polydisperse, as indicated by the high PDI values of 0.9 and 0.7, respectively. However, the PDI decreased to 0.5 for ALSOK2 and PGG, and a monodisperse distribution (PDI = 0.1) was evident for Brewtan F.

It is conceivable that the differences in the abilities of the gallotannins to promote mineralization of hydrogels may be linked with interactions between ALP and gallotannins. Mango extract, PGG, and Brewtan F were the most successful at promoting mineralization, while ALSOK2, and in particular ALSOK4, promoted mineralization to a markedly lower extent (Figure 1F,G). Interaction fluorescence quenching studies in interaction solution C, whose composition was closest to that of the hydrogels (the components ALP, CaCl_2_, and GG were in the same mass ratios as in the hydrogels), showed that PGG and Brewtan exerted the strongest ALP effect, while ALSOK2, and in particular ALSOK4, exerted markedly lower effects (Figure 3). Furthermore, PGG and Brewtan F caused formation of significantly larger ALP aggregates, with a diameter higher by over one order of magnitude (Table 2). Previous work has shown that ALP can diffuse out of GG hydrogels [2]. Larger aggregates of ALP would diffuse out of the hydrogels more slowly, leading to higher intrahydrogel concentrations of ALP and increased mineralization. Zeta potential measurements (Figure 4) showed that calcium makes zeta potential less negative, which would be expected to promote aggregation. No marked differences in zeta potential were observed between interaction solutions containing different gallotannins. Therefore, it can be speculated that the differences in aggregate size detected by DLS (Table 1) are not due to differences in zeta potential, but due to differences in gallotannin–ALP interactions (Figure 3), which might lead to increased protein diameter for PGG and Brewtan. It is not inconceivable that PGG and Brewtan exhibit higher affinities for Ca^2+^, leading to increased aggregation of gallotannin-Ca^2+^-ALP. 

The mango extract was not subjected to investigation due to the heterogeneity of its composition. It should be kept in mind that interaction solutions A, B, and C were diluted by a factor 10, so caution should be used in interpreting this data.

### 2.3. Cell Biological Characterization and Antibacterial Testing of Mineralized Hydrogels

The compatibility of mineralized gallotanin-enriched hydrogels with bone cells was evaluated by two approaches: (1) cultivation of human osteoblast-like Saos-2 cells in extracts (eluates) of the materials into the cell culture medium and (2) cultivation of Saos-2 cells directly on the materials. 

The growth of Saos-2 cells in eluates was evaluated using an xCELLigence system, which enables real-time monitoring of cell growth based on impedance generated by adhering cells.

**Figure 5:**
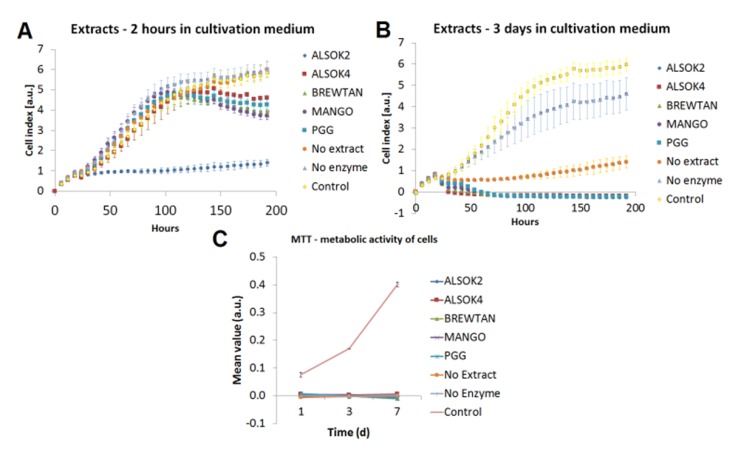
Cell biological and antibacterial testing. (**A**) Growth of Saos-2 cells in eluate from mineralized hydrogels containing different gallotannin preparations incubated for 2 h in cell culture medium. Tissue culture plastic served as a control. (**B**) Growth of Saos-2 cells in eluate from mineralized hydrogels containing different gallotannin preparations incubated for 3 d in cell culture medium. Tissue culture plastic served as a control. (**C**) Growth of Saos-2 cells on mineralized hydrogels containing different gallotannin preparations. Tissue culture plastic served as a control.

Cultivation of Saos-2 cells in eluates from mineralized hydrogels after 2 h incubation in cell culture medium (Figure 5A) revealed that samples with no extract and no enzyme displayed cytocompatibility similar to that of the control (cells grown in standard culture medium). Other samples showed poorer cytocompatibility after 150 h, with the exception of samples containing ALSOK2, which showed markedly poorer cytocompatibility from the start of the experiment.

Cultivation of Saos-2 cells in eluates from mineralized hydrogels after 3 d incubation in cell culture medium (Figure 5B) revealed that samples with no enzyme displayed the best cytocompatibility, which was, however, markedly worse than that of the control. Values for samples containing no extract were markedly lower still. All samples containing extracts displayed very poor cytocompatibility.

One explanation for the poor cell growth may be the toxic effect of DMSO [37]. However, in the mentioned study performed on Caco2/TC7 tumor cells, DMSO was used in relatively high concentrations ranging from 30% to 100%, while the 10% DMSO did not cause any cytotoxic effect, as revealed by assays of lactate dehydrogenase release and neutral red uptake. In addition, in 10% concentration, DMSO is currently used as a protective agent for cryopreservation of cells, including Saos-2 cells and bone marrow mesenchymal stromal cells, in which it preserved a high viability [38]. On the other hand, DMSO is known as an inhibitor of cell proliferation by arresting the cells in the G1 phase of the cell cycle, but the cell cycle was completely restored after the DMSO removal [39].

It is possible that release of calcium out of mineralized hydrogels may have killed cells. Calcium ion levels above 10 mM have been reported to be cytotoxic [40]. On the other hand, calcium-containing materials, such as calcium phosphate ceramics, can deplete calcium from the culture medium, which can significantly attenuate the cell proliferation [41,42]. This calcium depletion might also occur in our study, because self-mineralizing materials are logically active in capturing Ca ions from their surrounding environment. The growth of Saos-2 cells directly on the samples was evaluated by a MTS assay of the activity of cell mitochondrial enzymes. The MTS test (Figure 5C) revealed very poor growth on all hydrogel samples at all time points. This finding was unexpected, since previous work has shown that bone-like MC3T3-E1 and MG63 cells are able to adhere to the surfaces of enzymatically mineralized hydrogels [2,16,43].

However, in our previous work, viable MC3T3-E1 and MG-63 cells adhered and grew on enzymatically mineralized hydrogels, without the addition of tannins or other polyphenols. When phlorotannins were added to the hydrogels, these hydrogels became cytotoxic for osteoblast-like MG-63 cells [16]. Therefore, it can be supposed that the poor growth of osteoblast-like Saos-2 cells on our samples enriched with gallotannins or in extract from these samples was caused by cytotoxic effects of gallotannins. It has been also reported that gallotannins induced apoptosis, senescence, cell cycle arrest, and loss of the cell–cell adhesion in several human cell lines derived from colon cancer, breast cancer, prostate cancer, and hepatocellular carcinoma [44,45,46,47]. 

An interesting feature of tannins is certain selectivity in their cytotoxicity behavior towards tumor cells and normal cells. For this selectivity, gallic acid (specifically its carboxyl groups) is considered to be responsible [48]. Gallic acid derivatives were found to induce cell death in cancer cell lines but not in primary cultured rat hepatocytes and human keratinocytes [49]. Hydrolyzable tannins showed higher cytotoxic activity against human oral squamous cell carcinoma and salivary gland tumor cell lines than against normal human gingival fibroblasts [50]. Similarly, in our earlier study and our present study, both phlorotannins and gallotannins were cytotocxic towards MG-63 cells [16] and SaOs-2 cells (i.e., cells of osteosarcoma origin), while in a study by Karadeniz et al. [23] phlorotannins increased the growth, viability, and osteogenic cell differentiation in mouse MC3T3-E1 preosteoblasts, which are not of tumor origin. Thus, our further studies will focus on the effects of gallotannin-enriched hydrogels on primary human osteoblasts and human bone marrow derived mesenchymal stem cells. It may be worth considering applications for mineralized composites outside of the biomedical field, where cytocompatibility is less of an issue. For instance, the mineralization of hydrogels could possibly be useful in self-healing applications or environmental engineering applications to remove wastewater from unwanted metal ions, but detailed discussion is outside the scope of this paper.

### 2.4. Antibacterial Testing

**Figure A2:**
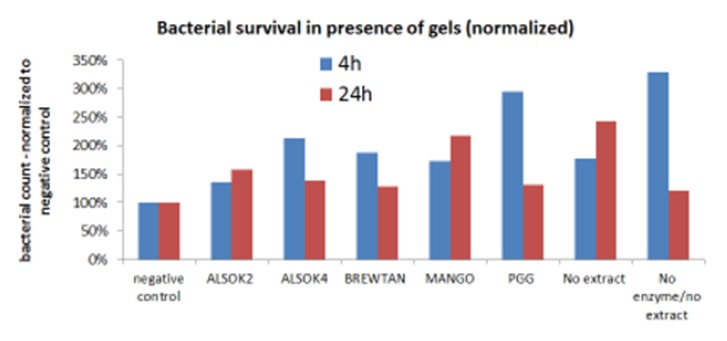
Survival of *E. coli* in the presence of growth of mineralized hydrogels containing different gallotannin preparations after 4 (blue) and 24 h (red). In all cases, *n* = 1.

The antibacterial activity of gallotannin-enriched hydrogels was tested using *Escherichia coli*, a model microorganism currently used for various experimental studies. The results of antibacterial testing (Figure A2) revealed no antibacterial effect after 4 h and 24 h. One explanation may be that gallotannins diffused out of the hydrogel during the mineralization process, and as a consequence, the amount of gallotannin remaining was too low to impede bacterial growth. Another explanation may be that the presence of mineral in the mineralized hydrogels or the non-covalent interaction with the ALP impedes diffusion of gallotannins to the surface, so insufficient amounts of gallotannin reach the bacteria. Another reason could be that the gallotannins show different antibacterial activities towards different bacterial species. In a study by Engels et al. [51], gallotannins did not inhibit the growth of lactic acid bacteria but only reduced the growth of Gram-negative *Escherichia coli*, and fully prevented the growth of Gram-positive food spoilage bacteria.

## 3. Materials and Methods

### 3.1. Materials

All materials, including GG (Gelzan™ CM, Product no. G1910, “low-acyl”, molecular weight 200–300 kD), ALP (bovine intestinal mucosa-derived, product no. P7640), and CaGP (50043), were obtained from Sigma-Aldrich, unless stated otherwise. PGG and three tannic acids (ALSOK2 (1040 D, 20% PGG), ALSOK4 (850 D), and Brewtan F (1450 D, 5% PGG)) were obtained from Aji OmniChem NV, Wetteren, Belgium. Extract from mango kernel was obtained as described previously [20,51].

### 3.2. GG hydrogel Production, Extract, and Enzyme Incorporation and Mineralization

GG hydrogels were prepared according to the method described previously [2]. GG powder (0.42 g) was sterilized under ultraviolet (UV) light for 2 h. A stock solution of GG was prepared by dissolving the sterilized GG powder in sterile distilled water (48 mL) preheated to 70 °C. A stock solution of CaCl_2_ (113.65 mg in 50 mL H_2_O) was sterilized by autoclaving (121 °C) and preheated to 70 °C. This CaCl_2_ stock solution was used as a crosslinking solution. ALP stock solution (250 mg in 10 mL H_2_O) was sterilized by filtration (0.2 µm, Cellulose filter) and stored at 4 °C in the dark. Gallotannin stock solutions were prepared by dissolving each gallotannin preparation or extract in dimethyl sulfoxide (DMSO) at a concentration of 25 mg/mL and sterilizing by filtration. These 4 stock solutions (GG, CaCl_2_, ALP, gallotannin) were mixed in 6-well plates under sterile conditions (3 mL GG, 0.66 mL CaCl_2_, 0.66 mL ALP, 0.66 mL gallotannin). After solidification, sterile hole punches were used to cut out disc-shaped samples. “No extract” hydrogels (containing pure DMSO with no gallotannins) and “no enzyme” hydrogels (containing pure DMSO without gallotannins and distilled water instead of ALP solution) served as controls. For mineralization studies, hydrogel disc samples of diameter 6 mm were cut out and immersed in 10 mL mineralization medium (CaGP, 4.2 g in 200 mL H_2_O, sterilized in autoclave) for 4 days.

### 3.3. Physicochemical Characterization of Mineralized hydrogels: Dry Mass Percentage, ICP-OES, SEM, XRD, FTIR

Hydrogels were dried at 60 °C for 72 h before physicochemical characterization to remove water. Dry mass percentage (i.e., the mass percentage of mineralized hydrogels attributable to polymer and mineral and not water) served as a measure of the extent of mineralization and was calculated as weight after mineralization before drying/weight after mineralization after drying x 100%. ICP-OES was performed as described before [52]. SEM was performed with a MIRA II LMU (Tescan, Brno, Czech Republic) at 20 kV in secondary electron mode. Prior to analysis, a drop of an aqueous suspension of the powder was air-dried on a silicon wafer at 22 °C. Powder XRD analysis of the polycrystalline samples was performed with a Rigaku Miniflex-600 diffract meter (Rigaku Corporation, Tokyo, Japan). The XRD data were recorded using Cu-Kα radiation (40 kV, 15 mA, Ni-Kβ filter) in the 2θ range of 5–60° at a scan speed 1°/min. The crystalline phases were identified with the use of integrated X-ray powder diffraction software (PDXL: Rigaku Diffraction Software) and The International Centre for Diffraction Data Powder Diffraction File (ICDD PDF)-2 datasets (Release 2014 RDB). The XRD data obtained were compared with the literature-based crystallographic data for hydroxyapatite (ref: 01-084-1998) [53]. FTIR was performed as described previously [54].

### 3.4. Interactions between Gallotannins and ALP

The interaction of different gallotannins with ALP was observed by using similar concentration ratios of the single compounds as those in the hydrogels. Stock solutions of GG, CaCl_2_, and ALP were prepared as described above but without sterilization. Here, 1 mg/mL of the gallotannins were dissolved in DMSO. Three different interaction solutions were prepared according to Table 1.

In the first interaction solution A the ALP was dissolved in water, in the second interaction solution B the ALP was dissolved with CaCl_2_ in water, and the third interaction solution C ALP was dissolved with CaCl_2_ and GG in water.

Non-covalent interactions between gallotannins and ALP were followed by fluorescence quenching analysis. Here, 1 mL of each interaction solution A, B, or C was further diluted by a factor of 10 to achieve an ideal fluorescence signal of the ALP. Here, 2 mL of each solution was filled in a quartz cuvette with four polished sides and the fluorescence emission at 340 nm was recorded at the excitation wavelength of 294 nm (using a Varian Cary Eclipse spectrometer, Varian Australia PTY. Ltd.). In addition, fluorescence spectra were recorded between 300 and 500 nm wavelengths at 294 nm emission against pure water as the reference. The same cuvettes were then placed in a UV-spectrometer (Beckmann Spectrophotometer DU530, Life Science UV/VIS) and the absorption at 294 and 340 nm wavelengths was measured against water for inner filter corrections. Following this, 10 µL of DMSO or gallotannins in DMSO (1 mg/mL) were added to the cuvette, stirred, incubated for 5 min, and fluorescence as well as UV absorption were recorded. Afterwards, a further 10 µL of the respective solutions were added and fluorescence and UV-absorption were measured until a maximum of 50 µL was reached. In this way, by adding 10, 20, 30, 40, and 50 µL gallotanin solution, ALP/gallotannin mass ratios of 76:1 38:1, 25.3:1, 19:1, and 15.3:1 were achieved (This corresponds to approximate ALP/gallotannin molecular weight ratios between 0.5:1 and 0.1:1). The saturation of ALP with bound gallotannins was achieved within this range.

Protein cross-linking was also assessed by size changes via dynamic light scattering (DLS) and electrostatic complexes were evaluated using zeta-potential measurements before and after ligand addition (Table 2).

At the beginning and end of the measurement, the zeta potential and size were recorded using a Malvern Zetasizer Nano ZS (Malvern Instruments GmbH, Herrenberg, Germany). The refractive index for proteins was 1.45, while that of water was taken to be 1.33. The viscosity of water at room temperature was taken to be 0.8872 cps (centipoise) for the samples.

A solution of 1 mL CaGP (210 mg/10 mL) was added to the final solutions of the ALP with gallotannins to assess the effect on the zeta potential. 

### 3.5. Cell Biological Characterization

#### 3.5.1. Preparation of Hydrogels for Direct Cell Seeding and Production of Eluates

After the mineralization, hydrogels were transferred into 10 mL of phosphate buffer saline (PBS) for 3 days to optimize pH. Hydrogels were then immersed in 10 mL of full cultivation medium (McCoy’ 5A). Three ml of the medium was taken as eluate after 2 h and another 3 mL was taken as eluate after 3 days. Eluates were used for real-time monitoring of cell growth in the xCellingence^®^ system; hydrogels were used for the direct cell seeding.

#### 3.5.2. Real-Time Monitoring of Cell Adhesion and Proliferation in Eluates

Cellular response of osteoblast-like Saos-2 cells (purchased from European Collection of Cell Cultures, Salisbury, UK) to different tannin acid eluates were studied at 37 °C in a humidified air atmosphere containing 5% of CO_2_ for 192 h. Cells were cultured in McCoy 5A medium containing fetal bovine serum (15%) and gentamicin (40 μg/mL). A real-time cell analyzer (xCelligence, Roche Applied Science, Mannheim, Germany) was used to evaluate the growth of cells in the prepared solutions continuously during an 8-day time span. The cells were seeded into 96-well sensory E plates (E-Plate 96, BioTech a.s., Prague, CR, Cat. No. 05232368001), and the background impedance was measured in each well. The cell density was 3500 cells/well (approximately 10 300 cells/cm^2^). The final volume of the medium with suspended cells was 200 μL. After 24 h, when the cells were attached to the well bottoms, the cultivation medium was exchanged for eluates taken after 2 h and 3 days. Each sample was added to the wells in quadruplicates. Cell on tissue culture plastic served as controls. The medium and eluates without cells served as negative controls. Cell index values (reflecting cell attachment, spreading, and proliferation) were calculated automatically by the instrument according to the formula: cell index = (impedance at individual time interval—background impedance)/15Ω).

As the primary goal of this study was to evaluate the cytocompatibility of mineralized gellan gum hydrogels loaded with different types of polyphenols, only loaded hydrogels were tested. The comparison of the hydrogel extracts with pure polyphenols extracts would have had limited value, as the extracts were solutions in pure DMSO, while the hydrogels contained a much smaller amount of DMSO and were incubated in mineralization solution for several days, lowering the DMSO concentration further.

#### 3.5.3. Evaluation of Cellular Growth on Hydrogels after Direct Seeding by MTS Test

Hydrogels (6 mm in diameter) were placed into 48-well plates and seeded with Saos-2 cells. Tissue culture plastic served as a control. Cells (density 18 620 cells/well, approximately 19 600 cells/cm^2^) were cultured in McCoy 5A medium containing fetal bovine serum (15%) and gentamicin (40 μg/mL). On days 1, 3, and 7, the cell viability was estimated by a test based on MTS tetrazolium (K300-500, BioVision) conversion. Briefly, a stock solution of MTS reagent (0.1 mL) was added to the medium (1 mL). Then, 1 mL of the solution was added to the cells washed with PBS in order to remove the former medium. After 2.5 h incubation at 37 °C and 5% CO_2_, the absorbance was measured (490 and 650 nm) and was corrected to the background control (a solvent mixture without cells) on a Synergy™ HT Multi-Mode Microplate reader (BioTek, USA).

### 3.6. Antibacterial Testing

*E. coli K12* was grown in LB medium (37 °C) to achieve an optical density (O.D.) at 450 nm of 0.5, which corresponded to approximately 10^8^ bacteria/mL, and then diluted in PBS buffer by a factor of 100 to obtain a concentration of approximately 10^6^ bacteria/mL. Hydrogels of 12 mm diameter were incubated with 3 mL of bacterial suspension at 37 °C with shaking at 150 rpm. Specific volumes of suspension were taken, properly diluted, applied on agar plates, and incubated for 24 h at 37 °C. A drop test was conducted, involving application of 5 µL from each dilution onto agar and comparison of the density of the spots between the negative control and the sample. This served as a pilot test. This was followed by a plate count, involving application of 100 µL of appropriately diluted suspension onto an agar plate. The colonies that grew were counted and counts were compared to the negative control. Experiments were performed once.

### 3.7. Statistical Analysis

If not stated otherwise, all sample solutions were prepared in triplicate. Statistical significance at a level of 5% was tested by analysis of variance (ANOVA) and Tukey´s post-hoc test with GraphPad Prism software (version 6.07, GraphPad Software, San Diego, USA).

## 4. Conclusions

Incorporation of a gallotannin-rich mango extract and preparations of PGG and tannic acid into GG hydrogels promoted enzymatic mineralization. Hence, gallotannins and ALP had synergistic effects on gellan gum mineralization, which could be exploited to produce composite biomaterials to replace irreversibly damaged bone tissue and also actively promote bone regeneration. The increase in mineralization was highly dependent on the gallotannin preparation. It was found in our studies that gallotannin–ALP interactions are dependent on the medium in which the interactions take place. Mineralized hydrogels containing gallotannins displayed reduced cytocompatibility and did not exhibit antibacterial activity towards *E. coli*.

## Figures and Tables

**Table 1:** Composition of interaction solutions used to study interactions between gallotannins and ALP.

Interaction Solution Name	ALP Stock Solution (mL)	Water (mL)	CaCl_2_ Stock Solution (mL)	GG Stock Solution (mL)	Final Volume (mL)
A	0.66	3.66	0	0	4.32
B	0.66	3	0.66	0	4.32
C	0.66	0	0.66	3	4.32

**Table 2:** Dynamic light scattering (DLS) measurements (z-average and polydispersity index (PDI)) of ALP aggregates in interaction solution C (see Table 2). In all cases, *n* = 3.

Interaction Solution	20 µL Interaction Solution *		50 µL Interaction Solution *	
	z-average (nm)	PDI	z-average (nm)	PDI
C (ALP/GG):DMSO (no gallotannins)	82 ± 15 ^a,b^	0.9	100 ± 32 ^1^	0.9
C (ALP/GG):ALSOK 4	80 ± 13 ^a^	0.9	91 ± 02 ^1^	0.7
C (ALP/GG):ALSOK 2	127 ± 08 ^a,b^	0.6	148 ± 19 ^1,2^	0.5
C (ALP/GG):PGG	118 ± 16 ^a,b^	0.6	166 ± 56 ^2^	0.5
C (ALP/GG):Brewtan F	150 ± 08 ^b^	0.2	207 ± 03 ^2,3^	0.1

Note: * 20 or 50 µL gallotannin solution (1 mg/mL) or pure DMSO (0 mg gallotannins/mL) was added to each interaction solution. All measurements were conducted in triplicate. The values are listed as mean ± standard deviation. Values with different superscripted letters or numbers are significantly different (<0.05). Values with the same number or letter are not significantly different.

## Abbreviations

ALP	Alkaline phosphatase
BTF	Brewtan F
CaGP	Calcium glycerophosphate
CaP	Calcium phosphate
CDHA	Calcium-deficient hydroxyapatite
DLS	Dynamic light scattering
DMSO	Dimethyl sulfoxide
FTIR	Fourier-Transform Infrared spectroscopy
GG	Gellan gum
ICP-OES	Inductively Coupled Plasma Optical Emission Spectroscopy
O.D.	optical density
PBS	phosphate buffer saline
PDI	polydispersity index
PGG	Pentagalloyl glucose
SEM	Scanning Electron Microscopy
Trp	tryptophan
UV	ultraviolet
XRD	X-Ray Diffraction
